# Comparison of lip repositioning and lip repositioning with botulinum toxin application in gummy smile treatment

**DOI:** 10.1016/j.jobcr.2026.101422

**Published:** 2026-02-13

**Authors:** Mert Keles, Dilek Ozkan Sen, Elif Oncu

**Affiliations:** Department of Periodontology, Faculty of Dentistry, Necmettin Erbakan University, Konya, Turkey

**Keywords:** Botulinum toxin, Esthetic dentistry, Gummy smile, Lip repositioning surgery, Smile design

## Abstract

**Background:**

This study aimed to compare the effects of lip repositioning surgery alone versus its combination with botulinum toxin type A injection in reducing gingival display and to evaluate the esthetic stability of both approaches over a 1-year follow-up.

**Methods:**

A retrospective analysis was conducted using clinical records of 29 patients with excessive gingival display. Group A (n = 14) underwent lip repositioning surgery alone, while Group B (n = 15) received the same surgery followed by botulinum toxin injection. Gingival visibility was assessed from archived clinical records at baseline and at 1, 3, and 12 months postoperatively, with available clinical photographs reviewed solely as supplementary documentation. Statistical analyses were performed according to data distribution.

**Results:**

Both groups showed significant within-group reductions in gingival display (p < 0.001). At 3 months, Group B demonstrated a significantly greater improvement (p < 0.001), indicating a short-term additive effect of botulinum toxin. However, by 12 months, the difference between groups was not statistically significant (p = 0.134), and surgical outcomes remained stable.

**Conclusion:**

Within the limitations of a retrospective design and the variability of clinical records, lip repositioning surgery provided predictable and long-term esthetic results in managing gummy smile. While botulinum toxin may enhance short-term outcomes, its temporary effect limits long-term benefits. Treatment planning should consider both objective outcomes and patient esthetic expectations.

## Graphical abstract

**Figure:**
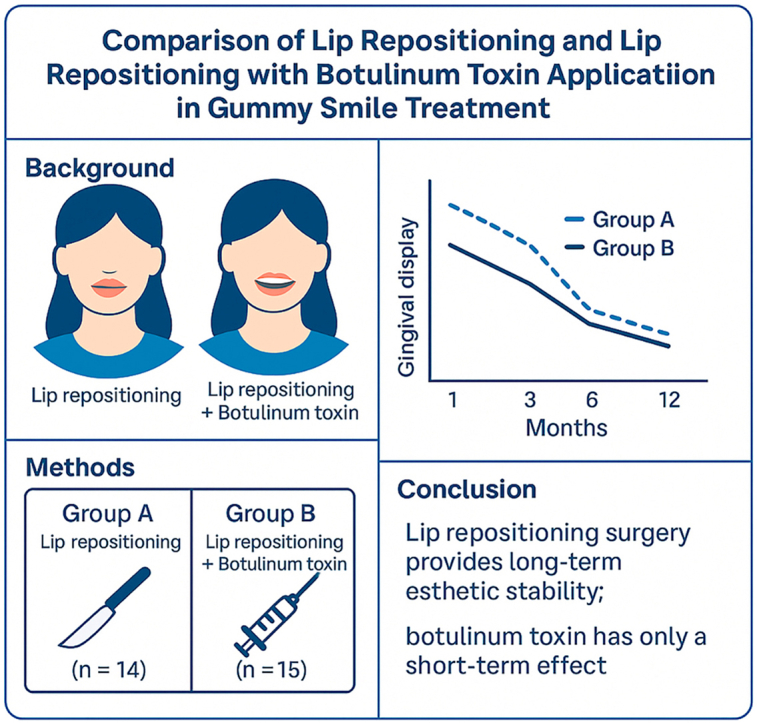

## 1 Introduction

Facial esthetics play a critical role in social perception and individual self-confidence, with the smile being one of the most influential elements.^1^ One of the key determinants of smile esthetics is the extent of gingival display during smiling. Ideally, a pleasing smile reveals all maxillary teeth and approximately 1–2 mm of gingival tissue. Gingival display exceeding 3 mm is generally perceived as unesthetic and is referred to as a “gummy smile” (GS).^2^^,^^3^

GS affects approximately 10.5% to 29% of the population and is reported more frequently in females.^4^ Beyond its esthetic implications, GS may negatively influence self-esteem, social interactions, and oral health-related quality of life, leading many patients to seek corrective treatment. The condition may result from various intraoral and extraoral etiologies, including vertical maxillary excess, a hypermobile or short upper lip, and altered passive eruption.^5^^,^^6^ Among these, hypermobile upper lip is frequently reported as a major contributing factor in a significant portion of patients seeking treatment.^7^ Accurate diagnosis of the underlying etiology is essential to select the most appropriate and effective treatment modality.^8^ However, from a clinical perspective, uncertainty remains regarding the optimal management strategy for gummy smile associated with hypermobile upper lip, particularly in terms of treatment stability and durability of esthetic outcomes.

Multiple approaches have been proposed to manage GS, such as orthognathic surgery,^9^ gingivectomy,^10^ botulinum toxin injection,^11^ and lip repositioning surgery.^12^^,^^13^ Lip repositioning, first introduced by Rubinstein and Kostianovsky, is a conservative surgical technique aimed at reducing gingival display by limiting the vertical movement of the upper lip.^14^ Over time, various modifications have been developed to improve its efficacy and minimize relapse, including techniques that preserve the labial frenulum or combine the procedure with crown lengthening.^15^^,^^16^ Although lip repositioning surgery is considered a successful method for GS treatment, its long-term effect is controversial. Studies showing that the long-term effect is uncertain or recurrent exist,^10^^,^^16^ as well as studies showing that it remains stable.^17^^,^^18^

In recent years, botulinum toxin type A has gained popularity as a minimally invasive option for GS correction, especially in cases associated with hypermobile upper lip.^19^ While it offers immediate esthetic improvement with fewer complications, its temporary nature necessitates repeated injections for long-term results.^20^ From a public health perspective, the growing demand for minimally invasive esthetic procedures highlights the need for evidence-based evaluation of their effectiveness and stability.

Despite the widespread use of lip repositioning surgery and botulinum toxin type A for the management of excessive gingival display, direct comparative evidence regarding their short- and long-term esthetic stability remains limited. In particular, data evaluating the durability of combined approaches beyond the early postoperative period are scarce. Moreover, existing studies often imply comparative superiority despite being based on retrospective designs. Therefore, the present retrospective study aimed to observe clinical trends and esthetic stability over a 12-month follow-up rather than to establish treatment superiority. The primary objective was to compare changes in gingival display between lip repositioning surgery alone and lip repositioning combined with postoperative botulinum toxin application at predefined time points. The secondary objective was to evaluate the temporal pattern and stability of treatment outcomes within each group. Although the effect of botulinum toxin is known to be temporary, its adjunctive use may influence early wound maturation and short-term esthetic outcomes. From a clinical decision-making perspective, understanding the magnitude and duration of this short-term benefit may assist clinicians in selecting individualized treatment strategies based on patient expectations, timing demands, and desired outcome longevity.

## 2 Materials and methods

### 2.1 Study design and patient population

This retrospective comparative observational study was conducted in accordance with the Strengthening the Reporting of Observational Studies in Epidemiology (STROBE) guidelines. A completed STROBE checklist is provided as supplementary material to ensure transparent reporting. Ethical approval was obtained from the relevant institutional ethics committee. The requirement for informed consent was waived due to the retrospective use of anonymized patient data. All procedures adhered to the principles of the Declaration of Helsinki (2013 revision). Clinical trial registration: not applicable.

Clinical records of patients treated for excessive gingival display at a university-based dental faculty between 2021 and 2023 were reviewed. To reduce selection bias inherent in retrospective designs, records were limited to patients with complete periodontal records and follow-up data documented by calibrated examiners. Baseline variables (age, gender, baseline gingival display) were compared between groups.

The study included 29 patients aged 18–65 years. Inclusion criteria were absence of systemic diseases, smoking fewer than 10 cigarettes per day, at least 20 remaining teeth, no active periodontal disease, and the presence of a hypermobile upper lip. For this retrospective analysis, hypermobility was defined as an upper lip elevation of ≥8 mm from rest to a posed maximum voluntary smile, based on archived clinical records and in accordance with previously published clinical criteria.^11^ Patients who were periodontally non-compliant or pregnant were excluded. Cases with incomplete follow-up data were excluded from the analysis. No imputation was performed for missing values.

A sample size estimation was performed using G∗Power 3.1 software to support the adequacy of the available sample size. Based on the effect size observed in a pilot group comparing gingival display reduction at 3 months, a minimum of 12 patients per group was calculated to detect a significant difference with a power of 80% and an alpha level of 0.05.

Patients were divided into two groups:

• **Group A (n** = **14):** lip repositioning surgery alone
• **Group B (n** = **15):** lip repositioning surgery combined with botulinum toxin type A injection

### 2.2 Preoperative procedures

Records were eligible for inclusion if they had been documented by two periodontists who had previously been calibrated in performing periodontal measurements. Examiner calibration was conducted prior to data extraction using 10 randomly selected patient records. An intraclass correlation coefficient (ICC) value ≥ 0.75 was considered acceptable, and the observed ICC of 0.88 indicated good inter-examiner reliability.

**Fig. 1:**
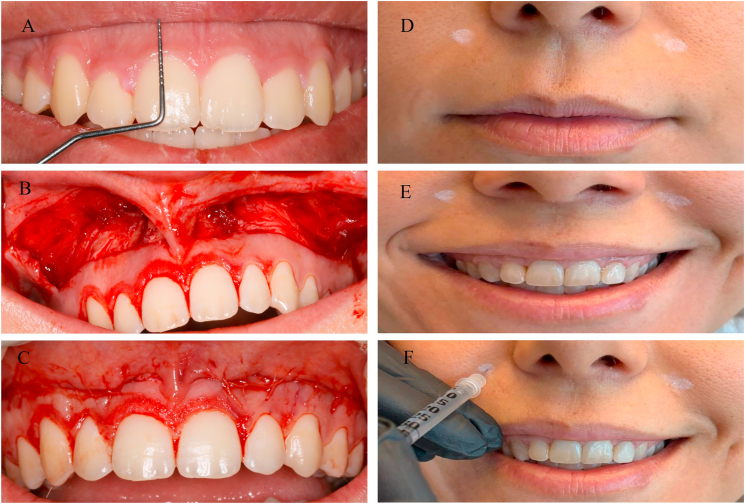
Clinical images related to surgical and botulinum toxin application protocols. A) Measurement of gingival visibility using a periodontal probe, B) View of the surgical site during upper lip repositioning surgery, C) Suturing applied following primary closure of the surgical site, D) Patient appearance in the resting position prior to botulinum toxin injection, E) Upper lip position of the patient during active smiling prior to botulinum toxin injection, F) The moment of botulinum toxin injection.

Where available, preoperative and follow-up photographs were retrospectively reviewed as supportive documentation. Owing to the retrospective design, photographic records were not consistently standardized in terms of lighting conditions, head position, or camera angle; therefore, photographs were considered supplementary only. Gingival display values were primarily extracted from archived clinical measurement records, which documented measurements obtained at the midline of the maxillary central incisors using a Williams periodontal probe (Hu-Friedy, USA). According to the clinical records, measurements were documented during a posed maximum voluntary smile, with patients seated in an upright position and the head in a natural posture. Smile elicitation was recorded as voluntary and non-forced. When available, recorded measurements were visually cross-checked against frontal images to verify consistency (Fig. 1A). Due to the retrospective nature of data acquisition, minor variations in head posture and smile elicitation could not be fully controlled; however, all measurements were derived from records documented by calibrated examiners under routine clinical conditions.

In cases of discrepancies between record entries, values were resolved through record reconciliation; when resolution was not feasible, averaged values were used. The reliability of the data extracted from archived records was assessed using intraclass correlation coefficients (ICC).

### 2.3 Surgery protocol

According to the archived surgical records, oral antisepsis with 0.12% chlorhexidine gluconate was performed prior to surgery in both groups. Local anesthesia was documented as having been administered to the vestibular mucosa and upper lip region between the first molars using 2% lidocaine with 1:100,000 epinephrine.

When required for esthetic alignment, minor gingivoplasty was documented as part of the routinely applied surgical approach to harmonize gingival contours. Surgical records indicated that, during lip repositioning surgery, incision margins were determined using a periodontal probe. A partial-thickness horizontal incision was recorded as extending from the mesial aspect of the left maxillary first molar to the mesial aspect of the right maxillary first molar, approximately 1 mm coronal to the mucogingival junction. A second parallel horizontal incision was documented 10–12 mm apical to the first incision, and the two incisions were connected at the molar line angles, creating an elliptical outline (Fig. 1B). Preservation of the labial frenulum was noted in all cases.

The intervening mucosal strip was documented as having been excised by superficial split-thickness dissection, leaving the underlying connective tissue exposed. Closure was recorded as being achieved using a continuous locked suture with 5-0 polyglycolic acid (Pegelak®, Doğsan, Turkey), with attention to midline preservation (Fig. 1C).^21^

### 2.4 Botulinum toxin application procedure

According to clinical records archived, only patients who received botulinum toxin type A 14 days after lip repositioning surgery were included in Group B. The records indicated that injections were administered at the Yonsei points, located approximately 1 cm lateral to the nostrils and 3 cm above the corners of the mouth. Each point received 3 units of onabotulinum toxin A (Botox®, Allergan Inc., USA), resulting in a total dose of 6 units per patient. All injections were documented as having been performed under sterile conditions and in accordance with the standard dosage protocol routinely used in the clinic.^11^

### 2.5 Postoperative protocol

Patients were prescribed analgesics (Dexketoprofen, 25 mg, three times daily for one week), antibiotics (Amoxicillin, 1000 mg, twice daily for one week), and chlorhexidine mouthwash twice daily for two weeks. Postoperative instructions included applying ice packs to the upper lip area during the first 24 h, avoiding mechanical trauma, and minimizing excessive lip movements while smiling or speaking for two weeks.

**Fig. 2:**
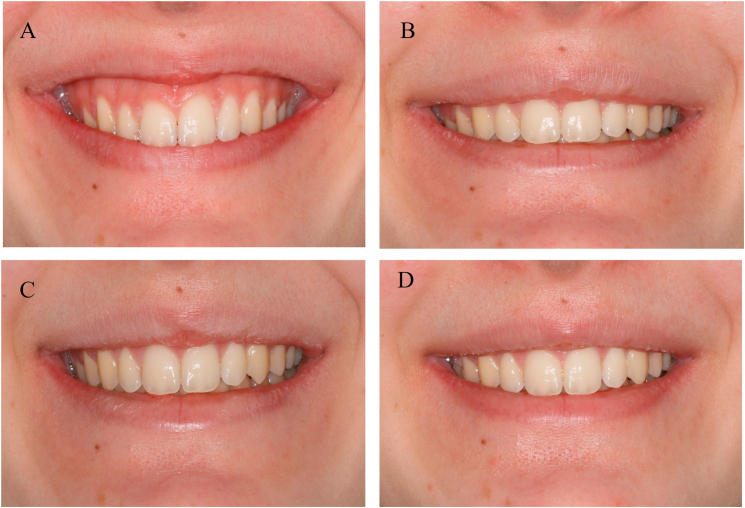
Changes in gingival visibility over time in Group A. A) Baseline, B) 1-month follow-up, C) 3-month follow-up, D) 12-month follow-up.

**Fig. 3:**
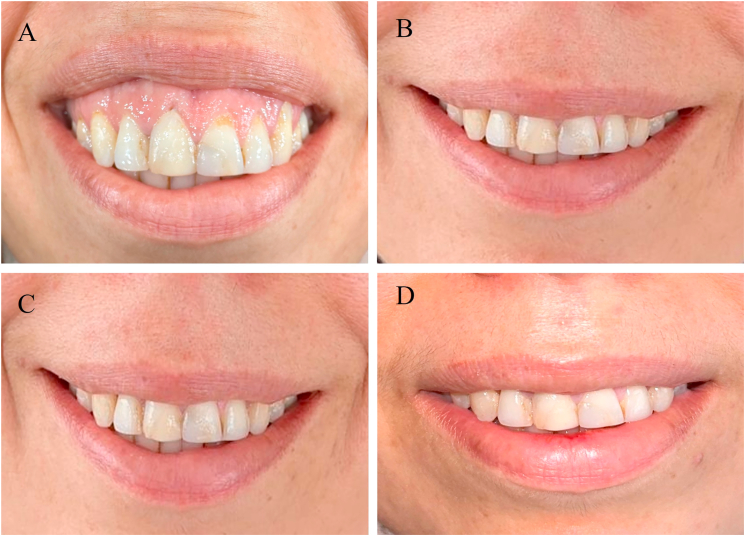
Changes in gingival visibility over time in Group B. A) Baseline, B) 1-month follow-up, C) 3-month follow-up, D) 12-month follow-up.

Assessments of gingival visibility at baseline and at 1-, 3-, and 12-month follow-ups were extracted from the archived clinical records documented by two calibrated periodontists. In cases of discrepancies in the records, consensus or averaged values were considered. Where available, preoperative and follow-up photographs were reviewed only as supplementary documentation to support the clinical records; however, due to the retrospective nature of the study, these photographs were not always standardized in terms of lighting, head position, or camera angle. Representative examples are shown in Fig. 2, Fig. 3.

### 2.6 Statistical analysis

Data were analyzed using IBM SPSS v27 (IBM Corp., Armonk, NY, USA). Normality and homogeneity of variances were assessed using the Shapiro–Wilk and Levene tests, respectively. Depending on the data distribution, between-group comparisons were performed using the independent samples *t*-test or the Mann–Whitney *U* test. Repeated measures were analyzed with the Friedman test followed by Bonferroni-adjusted post hoc analyses. Correlation analyses were conducted using Pearson or Spearman coefficients according to distribution characteristics. Fisher's exact test was applied for categorical variables when assumptions were not met. Effect sizes (Cohen's d) were calculated for between-group comparisons to evaluate the magnitude and clinical relevance of significant differences. Inter-observer reliability was assessed using intraclass correlation coefficients (ICC, two-way random, absolute agreement). An ICC value ≥ 0.75 was considered acceptable. Statistical significance was set at p < 0.05.

## 3 Results

A total of 29 participants were included in the study: 14 in Group A and 15 in Group B.

The gender distribution was comparable (Group A: 64.3% female; Group B: 66.7% female; p = 1.000), and no significant difference was observed in mean age (Group A: 27.21 ± 5.39 years; Group B: 30.20 ± 6.33 years; p = 0.184), confirming demographic homogeneity at baseline (Table 1).

**Table 1:** Baseline demographic characteristics of participants in Groups A and B.

Variable	Group A (n = 14)	Group B (n = 15)	Test Statistic	p-value
**Age (years)**	27.21 ± 5.39	30.20 ± 6.33	t = −1.363	0.184
**Gender**			Fisher's exact	1.000
Female	9 (64.3%)	10 (66.7%)		
Male	5 (35.7%)	5 (33.3%)		

Values are presented as mean ± standard deviation or number (%), as appropriate. Between-group comparisons were performed using the independent samples *t*-test for age and Fisher's exact test for gender due to small sample size.

**Fig. 4:**
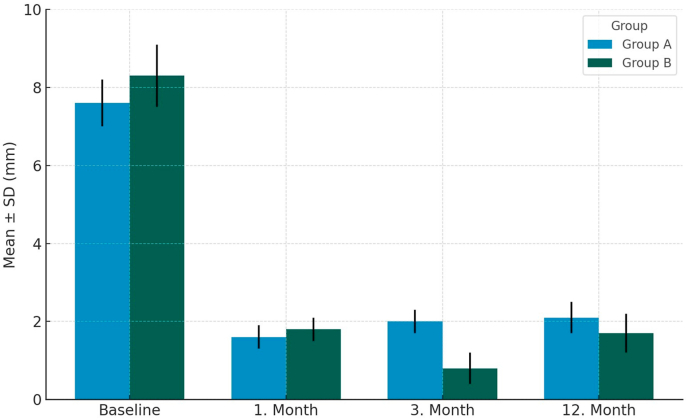
Changes over time in the mean (Mean ± SD, mm) values of gingival visibility in patients in Group A and Group B. The figure summarizes mean gingival display measurements at baseline and at 1-, 3-, and 12-month follow-up. A statistically significant between-group difference was observed at the 3-month follow-up (∗p < 0.001), corresponding to the peak effect of botulinum toxin application.

At baseline, the mean gingival visibility was 7.57 ± 1.28 mm in Group A and 8.20 ± 1.57 mm in Group B, with no statistically significant difference (p = 0.250) (Table 2, Fig. 4).

**Table 2:** Distribution and comparison of gingival visibility measurements according to measurement times for participants in groups A and B.

Time point	Group A Mean ± SD	Group B Mean ± SD	Test statistic (t or z)	p-value	Effect size (Cohen's d)
Baseline	7.57 ± 1.28	8.20 ± 1.57	z = −1.176†	0.250	0.44
1 month	1.64 ± 0.50	1.80 ± 0.68	t = −0.576	0.621	0.26
3 months	2.00 ± 0.55	0.53 ± 0.83	t = −3.969	<0.001∗	**2.10**
12 months	2.07 ± 0.62	1.60 ± 1.06	t = −1.637	0.134	0.54

Between-group comparisons were performed using the independent samples *t*-test or the Mann–Whitney *U* test, depending on data distribution. P-values are reported; exact values are provided where applicable. Effect sizes (Cohen's d) were calculated using pooled standard deviations to allow comparison of effect magnitude across time points, irrespective of the statistical test applied, and were interpreted as small (0.2), moderate (0.5), or large (0.8).

† Mann–Whitney *U* test (z value); ∗ statistically significant difference (p < 0.05).

These findings indicate that both groups presented with a similar esthetic profile characterized by moderate-to-severe gingival display, justifying surgical intervention.

At the 1-month follow-up, gingival display decreased significantly in both groups (Group A: 1.64 ± 0.50 mm; Group B: 1.80 ± 0.68 mm). No significant intergroup difference was detected (p = 0.621). This demonstrates that lip repositioning surgery effectively reduced gingival exposure in the early postoperative period, regardless of adjunctive botulinum toxin use.

At 3 months, a clear divergence was observed between the groups. Group A exhibited a slight relapse compared with the first month (2.00 ± 0.55 mm), while Group B showed further improvement (0.53 ± 0.83 mm). The between-group difference was statistically significant (p < 0.001) and corresponded to a large effect size (Cohen's d = 2.1). These results indicate that the addition of botulinum toxin provided a clinically relevant short-term enhancement in esthetic outcomes.

At the 12-month follow-up, a mild increase in gingival visibility was observed in both groups (Group A: 2.07 ± 0.62 mm; Group B: 1.60 ± 1.06 mm). However, the intergroup difference was no longer significant (p = 0.134). Although Group B maintained numerically lower gingival display values, the diminishing difference highlights the temporary nature of the botulinum toxin effect. Importantly, both groups sustained clinically acceptable levels of gingival display (<3 mm), reflecting long-term stability of the surgical outcome.

Within-group analyses confirmed significant changes in gingival visibility across all time points (Group A: χ^2^ = 34.324, p < 0.001; Group B: χ^2^ = 37.162, p < 0.001). The most pronounced intergroup difference was recorded at 3 months, underscoring the short-term benefit of botulinum toxin as an adjunctive procedure.

Following examiner calibration performed prior to data extraction (overall ICC = 0.88), time point–specific ICC values were calculated separately for each group. Inter-observer reliability of gingival display measurements extracted from archived clinical records was high across all assessment time points. For Group A, ICC values were 0.96 at baseline, 1.00 at 1 month, 0.77 at 3 months, and 0.82 at 12 months. For Group B, ICC values were 0.97 at baseline, 0.85 at 1 month, 0.90 at 3 months, and 1.00 at 12 months, confirming the robustness and reproducibility of the clinical data.

## 4 Discussion

This study tested the hypothesis that combining lip repositioning surgery with botulinum toxin type A would yield superior esthetic results at 3 months compared to surgery alone, but that this advantage would diminish at 12 months. The findings of this study support this hypothesis. At the 3-month follow-up, the combination group showed a significantly greater reduction in gingival display both statistically and clinically (Cohen's d = 2.1), while no significant difference was observed between the groups at 12 months. The 12-month follow-up period represents a relatively long observation window compared to much of the existing literature on gummy smile treatment, which often focuses on short-term outcomes, thereby allowing a more meaningful assessment of treatment stability over time. The inclusion of effect size analysis provides additional clinical context by quantifying the magnitude of this difference, thereby supporting interpretation beyond statistical significance alone. This model is consistent with the known pharmacodynamics of botulinum toxin, which typically shows effects in the short term and decreases over 6 months due to neuromuscular recovery.

Our findings are consistent with previous studies emphasizing the short-term efficacy and transient nature of botulinum toxin in the treatment of excessive gingival display.^1^^,^^3^^,^^4^ In contrast, studies evaluating lip repositioning alone have shown variable results; some authors reported stable long-term outcomes,^17^ while others noted moderate relapse.^16^ Our data show a slight increase in gingival visibility at 3 months, but overall stability within clinically acceptable limits (<3 mm) at 12 months.

An important strength and novel aspect of the present study is the standardized postoperative timing of botulinum toxin type A application following lip repositioning surgery. Previous studies have reported heterogeneous protocols regarding the timing of botulinum toxin administration, ranging from preoperative injection to delayed postoperative application, often without a clear rationale.^22^^,^^23^ In contrast, the present study applied botulinum toxin at a consistent postoperative time point, allowing a more reliable evaluation of its short-term adjunctive effect on gingival display. Most existing studies have primarily investigated the preoperative use of botulinum toxin as an adjunct to lip repositioning surgery. For example, a recent study demonstrated that botulinum toxin administered 15 days before surgery significantly reduced gingival display and improved the stability of 6-month results.^22^ However, postoperative administration, as evaluated in the present study, is designed to target the critical healing phase by potentially limiting muscle retraction during early wound.

Differences in results between studies may be due to differences in surgical technique, patient selection, or follow-up protocols. In our study, technical standardizations such as excluding the labial frenulum and using continuous locked sutures were applied to minimize the risk of recurrence. This is consistent with a recent study that highlights the importance of surgical consistency as a key factor in the predictability of outcomes, showing positive long-term results observed only in the lip repositioning group.^24^

From a clinical perspective, these findings underscore that treatment planning should be individualized based not only on objective outcomes but also on patient expectations and lifestyle. Botulinum toxin can provide meaningful esthetic gains in the short term, especially for patients seeking rapid improvement before social events, but its effects are temporary. In contrast, lip repositioning surgery offers more permanent results and may be preferred by patients aiming for long-term stability without repeated interventions.

This study has several limitations that should be considered when interpreting the results. First, the retrospective design inherently involves selection bias, as group allocation was non-random and based on available clinical records rather than prospective randomization, which limited control over data collection and standardization. Although the surgical and botulinum toxin application protocols were standardized and allow for procedural replicability, data reproducibility is limited by reliance on archived clinical records and non-standardized photographic documentation. Consequently, exact replication of measurement conditions may not be feasible. Second, the relatively small sample size may have reduced the statistical power to detect subtle intergroup differences at the 12-month follow-up, thereby increasing the risk of a type II error and potentially contributing to the absence of statistically significant long-term differences between groups. In addition, the single-center nature of the study may limit the external validity and generalizability of the findings to other clinical settings and patient populations. Third, the absence of patient-reported outcome measures (PROMs), such as Visual Analog Scale (VAS) scores, satisfaction ratings, or quality-of-life assessments, restricted the interpretation of patients’ perceived esthetic success.^25^ Furthermore, individual differences such as muscle anatomy or baseline muscle tone were not evaluated in relation to the effect of botulinum toxin. These factors should be considered in future prospective studies with larger sample sizes and combined subjective–objective outcome assessments.

Future research should focus on multicenter, prospective designs that integrate both clinical measurements and PROMs to capture patient-centered outcomes. Comparative studies assessing different timings of botulinum toxin injection (preoperative vs postoperative) and hybrid protocols combining minimally invasive esthetic interventions with surgery could also provide valuable insights. Moreover, exploring the public health relevance of gummy smile treatment, including its psychosocial impact and accessibility of minimally invasive esthetic procedures, would broaden the scope of clinical significance.

## 5 Conclusions

Within the limitations of this retrospective study and a 12-month follow-up period, lip repositioning surgery appears to be a predictable and clinically stable treatment option for excessive gingival display associated with a hypermobile upper lip. While adjunctive botulinum toxin type A can provide meaningful short-term esthetic improvement, its effects are not sustained in the long term. Treatment selection should therefore be individualized according to patient expectations, timing demands, and the desired duration of outcomes. Importantly, as a minimally invasive approach, lip repositioning represents an effective alternative to more radical surgical options such as orthognathic surgery, offering lower morbidity and faster recovery.

## Patient's/guardian's consent

Not applicable. This retrospective study used anonymized patient data.

## Ethical approval

Ethical approval was obtained from an institutional ethics committee.

## Sources of funding

This research did not receive any specific grant from funding agencies in the public, commercial, or not-for-profit sectors.

## Declaration of competing interest

The authors declare that they have no known competing financial interests or personal relationships that could have appeared to influence the work reported in this paper.
